# Genome Sequence of Corynebacterium ulcerans Strain FRC11

**DOI:** 10.1128/genomeA.00112-15

**Published:** 2015-03-12

**Authors:** Leandro de Jesus Benevides, Marcus Vinicius Canário Viana, Diego César Batista Mariano, Flávia de Souza Rocha, Priscilla Carolinne Bagano, Edson Luiz Folador, Felipe Luiz Pereira, Fernanda Alves Dorella, Carlos Augusto Gomes Leal, Alex Fiorini Carvalho, Siomar de Castro Soares, Adriana Carneiro, Rommel Ramos, Edgar Badell-Ocando, Nicole Guiso, Artur Silva, Henrique Figueiredo, Vasco Azevedo, Luis Carlos Guimarães

**Affiliations:** a Laboratory of Cellular and Molecular Genetics (LGCM), Federal University of Minas Gerais, Belo Horizonte, Minas Gerais, Brazil; b National Reference Laboratory for Aquatic Animal Diseases (AQUACEN), Ministry of Fisheries and Aquaculture, Federal University of Minas Gerais, Belo Horizonte, Minas Gerais, Brazil; c Laboratory of Polymorphic DNA (LPDNA), Federal University of Para, Belém, Brazil; d Institut Pasteur, Unité de Prévention et Thérapies Moléculaires des Maladies Humaines, National Centre of Reference of Toxigenic Corynebacteria, Paris, France

## Abstract

Here, we present the genome sequence of Corynebacterium ulcerans strain FRC11. The genome includes one circular chromosome of 2,442,826 bp (53.35% G+C content), and 2,210 genes were predicted, 2,146 of which are putative protein-coding genes, with 12 rRNAs and 51 tRNAs; 1 pseudogene was also identified.

## GENOME ANNOUNCEMENT

Corynebacterium ulcerans is a bacterium that presents catalase-positive, nitrate-negative, and urease-positive biochemical proprieties (1). This bacterium belongs to the Actinobacteria class, which includes the genera Corynebacterium, Mycobacterium, Nocardia, and Rodococcus, collectively termed the CMNR group. This is a very heterogeneous group; however, most of the species share particular characteristics, such as (i) a specific organization of the cell wall, which is mainly composed of peptidoglycans, arabinogalactans, and mycolic acids, and (ii) high G+C content (2–4).

Although C. ulcerans has increasing medical and veterinary importance, little is known about its lifestyle and associated virulence factors (5). The sequencing of more C. ulcerans genomes of both toxigenic and nontoxigenic strains will help in the identification of distinctive features of strains from human and animal sources (6). In addition, the data generated by newly sequenced genomes are helpful for identifying antibiotic and vaccine targets by way of a comparative analysis (7).

Nowadays, only seven complete genomes and two drafts are available in the National Center for Biotechnology Information (NCBI) database (http://www.ncbi.nlm.nih.gov/genome/). This scenario shows that more genomic knowledge is required in order to better characterize the virulence mechanisms of this emergent pathogen.

In the current study, we present the genome sequence of C. ulcerans strain FRC11, isolated from a 74-year-old human a with leg ulcerans infection in Toulouse, France. This strain was first identified as Corynebacterium pseudotuberculosis (8), but recent analysis shows that it belongs to C. ulcerans.

The sequencing, assembly, and annotation of this strain were performed by the teams from the Laboratory of Cellular and Molecular Genetics (LGCM) and the National Reference Laboratory for Aquatic Animal Diseases (AQUACEN), both located at the Federal University of Minas Gerais, Belo Horizonte, Minas Gerais, Brazil, and the Laboratory of Polymorphic DNA (LPDNA) at the Federal University of Pará, Belém, Pará, Brazil.

The platform used for sequencing was the Ion Torrent Personal Genome Machine (PGM) system (Life Technologies), using a fragment library. The quality of the raw data was analyzed using the Web tool FastQC (http://www.bioinformatics.babraham.ac.uk/projects/fastqc/). The assembly was done using the Simple Manager for Bacterial Assemblies (SIMBA) interface (http://ufmg-simba.sourceforge.net). The reads with good quality were assembled using a *de novo* strategy with the software MIRA 4.0 (9).

The assembly produced a total of 30 contigs, with a coverage of 179.14× and an *N*_50_ contig length of 236.335. Additionally, a scaffold was created using the CONTIGuator 2 software (10), using the genome sequence of C. ulcerans strain 0102 (accession no. NC_018101.1) (11) as a reference. The gap closure was performed automatically using SIMBA and manually using the CLC Genomics Workbench 7 software.

The genome was automatically annotated using Rapid Annotations using Subsystems Technology (RAST) (12). The manual curation of the annotation was performed using the Artemis software (13) and the UniProt database (http://www.uniprot.org). The CLC Genomics Workbench 7 software was used to correct indel errors in the regions of homopolymers.

The genome includes one circular chromosome of 2,442,826 bp (53.35% G+C content), and 2,210 genes were predicted, 2,146 of which are putative protein-coding genes, with 12 rRNAs and 51 tRNAs; 1 pseudogene was also identified.

### Nucleotide sequence accession number.

This genome has been deposited in GenBank under the accession no. CP009622.
